# Exploring the potential of herbal drugs for treating liver fibrosis: a computational screening approach

**DOI:** 10.2478/abm-2025-0010

**Published:** 2025-04-30

**Authors:** Tanya Ralli, Abdulsalam Alhalmi

**Affiliations:** 1 Department of Pharmaceutics, School of Pharmacy, COER University, Roorkee 247667, India; 2 Department of Pharmaceutics, Faculty of Pharmacy, University of Aden, Aden 8916162, Yemen

**Keywords:** liver fibrosis, molecular docking, pirfenidone, TGF-β, TNF-α

## Abstract

**Background:**

With the increasing prevalence of metabolic disorders such as obesity and hyperlipidemia, there is a heightened tendency for inflammation in the hepatocytes, which can eventually progress to liver fibrosis. Despite its high incidence, no approved treatment currently exists for liver fibrosis.

**Objectives:**

This study aims to identify potential herbal drugs with anti-fibrotic activity by targeting multiple pathways involved in liver fibrosis, particularly focusing on the Tumour growth factor-beta (TGF-β) and tumor necrosis factor-alpha (TNF-α) proteins.

**Methods:**

We conducted in silico studies on 9 widely used herbal drugs to evaluate their binding affinities for TGF-β and TNF-α receptors. The herbal drugs analyzed included ginseng, danshen, silymarin, resveratrol, berberine, anthocyanin, ginger, curcumin, and tocopherol.

**Results:**

Our results indicate that ginseng and danshen exhibit the strongest anti-fibrotic potential, with the most favorable binding energies for both TGF-β and TNF-α receptors. Silymarin, resveratrol, berberine, and anthocyanin also demonstrated comparable or superior activity to the reference drug and pirfenidone. Conversely, ginger, curcumin, and tocopherol showed relatively lower activity.

**Conclusions:**

Herbal drugs such as ginseng and danshen present promising candidates for the treatment of liver fibrosis due to their strong binding affinity to key fibrosis-related proteins and their lower side effect profile compared with synthetic drugs. The appropriate selection and combination of these herbal drugs could offer a viable therapeutic approach for managing liver fibrosis.

Liver fibrosis is one of the key manifestations associated with chronic liver damage. Various mediators, such as diet, alcohol, gut microbiota, metabolic-associated steatotic liver disease (MASLD) [1, 2], and obesity, lead to an inflammatory cascade in the liver [3]. Of all the liver cells, mainly hepatocytes, macrophages, and hepatic stellate cells (HSCs) cause the release of inflammatory cytokines like interleukin (IL)-6, Interleuking -1 beta IL-1β, and tumor necrosis factor-alpha (TNF-α). These cytokines in turn lead to inflammation in the liver. Moreover, the activation of HSCs further causes the release of cytokines related to fibrosis, mainly tumor growth factor-beta (TGF-β), α-smooth muscle actin (α-SMA), collagen I, and collagen III, which leads to the deposition of extracellular matrix in the hepatic cells which finally cause hepatic fibrosis [4].

Among the various cytokines involved in liver fibrosis, TGF-β and TNF-α play crucial roles in its pathogenesis [5, 6]. TGF-β is involved in all stages of disease progression, from inflammation to fibrosis, cirrhosis, and cancer, primarily by activating HSCs into myofibroblasts, which lead to significant hepatocyte death [7]. Besides its profibrogenic role, TGF-β also acts as a negative regulator of cell proliferation and an inducer of apoptosis [8], making it a primary target for developing anti-fibrotic therapies.

Similarly, TNF-α is an inflammatory cytokine that promotes liver inflammation, which can progress to fibrosis if unchecked [9]. TNF-α also contributes to apoptosis and necroptosis in liver cells [10]. A study by Osawa et al. [11] demonstrated that TNF-α promotes liver fibrosis through production of the tissue inhibitor of metalloproteinase (TIMP)-1 from HSCs, highlighting its importance in hepatotoxicity. Thus, targeting TGF-β and TNF-α presents a potential therapeutic approach for treating liver fibrosis.

Therefore, drugs acting by inhibiting either TGF-β or TNF-α or both can serve as a potential treatment option for treating liver fibrosis. Chemical drugs usually offer problems related to adverse reactions; thus, herbal drugs can act as a good therapeutic option for its treatment because they mainly show pharmacological action by acting on multiple pathways. Traditional herbal medicines (THMs), which are a prominent source of natural medicines or herbal drug products, are comprised of multiple biomarker molecules [12]. These THMs act by treating multiple organ–multiple hit models.

In selecting herbal ligands for this study, we compiled data from clinicaltrials.gov, focusing on herbal medicines currently in various phases of clinical trials for liver fibrosis and related conditions; of all the herbal medicines, mainly silymarin and berberine were in Phase 4 of clinical trials [13]. We selected 9 herbal ligands based on their traditional use for liver health and their potential to modulate pathways involved in liver fibrosis. Among these, silymarin, resveratrol, curcumin, and berberine have been extensively studied for their anti-fibrotic properties. Silymarin, in particular, is a well-known hepatoprotective agent containing multiple active compounds such as silybin A, silybin B, and isosilybin A, which have demonstrated efficacy in various liver disorders [14–17].

For comparison, pirfenidone was chosen as the standard synthetic compound due to its established efficacy in treating fibrotic conditions, including idiopathic pulmonary fibrosis and its potential off-label use in liver fibrosis. Pirfenidone has been shown to inhibit pro-fibrotic cytokines like TGF-β, reducing fibrosis progression in various models. By comparing the docking results of the selected herbal ligands with pirfenidone, we aimed to evaluate their binding affinities and potential therapeutic efficacy against a known anti-fibrotic agent [18–20].

## Methods

### Software and tools

The PubChem database was chosen due to its comprehensive collection of chemical structures and bioactivity data, which is crucial for accurate ligand preparation in molecular docking studies [21, 22]. Molecular docking was performed using AutoDock Tools 1.5.6 (Molecular Graphics Lab at The Scripps Research Institute, USA), and the protein structures were processed with the addition of polar hydrogen and Kollman charges. AutoDock is one of the most popular molecular docking software packages used for predicting how small molecules, such as drug candidates, bind to a receptor of known 3D structure. AutoDock Tools 1.5.6 provides a graphical user interface to set up docking simulations, including the preparation of protein and ligand files. The efficacy of AutoDock in virtual screening and molecular docking has been demonstrated in numerous studies [23, 24]. In addition to AutoDock, other software tools such as ChemDraw Professional 15.0 (Revvity Signals Software, a division of Revvity, Inc), MGL tools (Center for Computational Structural Biology (CCRB) at Scripps Research), OpenBabel (an open-source software project), Discovery Studio 2021 client (Dassault Systemes BIOVIA), and resources like the Protein Data Bank were utilized to enhance the accuracy and efficiency of molecular docking and structural analysis processes.

## Molecular docking study

### Ligands preparation

The 3D structures of all the selected ligands were downloaded from PubChem database in .sdf format (http://pubchem.ncbi.nlm.nih.gov). Furthermore, these files were converted into .pdb format using OpenBabel software. Autodock Tools 1.5.6 was used to convert .pdb files into. pdbqt format [25, 26]. The following were the herbal ligands selected for in silico antifibrotic activity, resveratrol [27], curcumin, berberine [28], silymarin [29–32], danshen, Panax ginseng, ginger, alpha tocopherol, and anthocyanin.

### Proteins preparation

Research Collaboratory for Structural Bioinformatics (RCSB) protein databank (http://www.rcsb.org) was used to download the structures of two target proteins, that is, TGF-β (PDB ID: 2WOT) [33] and TNF-α (PDB ID: 6RMJ) [34], for estimating the anti-fibrotic action. The enzyme structures were retrieved in .pdb format from the RCSB site. Further processing of these structures was done by first deleting the water molecules, then adding polar hydrogen molecules, and finally adding the Kollman charges in Autodock tools 1.5.6. The processed structures were then saved in. pdbqt format.

### Docking study using AutoDock 1.5.6

The energy minimized structures of ligand (in .pdbqt format) was docked by inserting it into the rigid protein molecule (in .pdbqt format) by using Autodock tools 1.5.6 [25]. For the purpose of blind docking, the entire structure of protein was enclosed inside a grid box with the grid spacing of 1 Å. To identify the pose with the lowest binding affinity, the protein structure was kept rigid, while the ligand molecule was kept flexible. The interaction energy between the ligand and receptor was expressed as affinity in kcal/mol and inhibition coefficient in μm. Discovery Studio 2021 Client was used to study the interactions between the ligand and the protein molecule.

## Results

**Figure 1.**
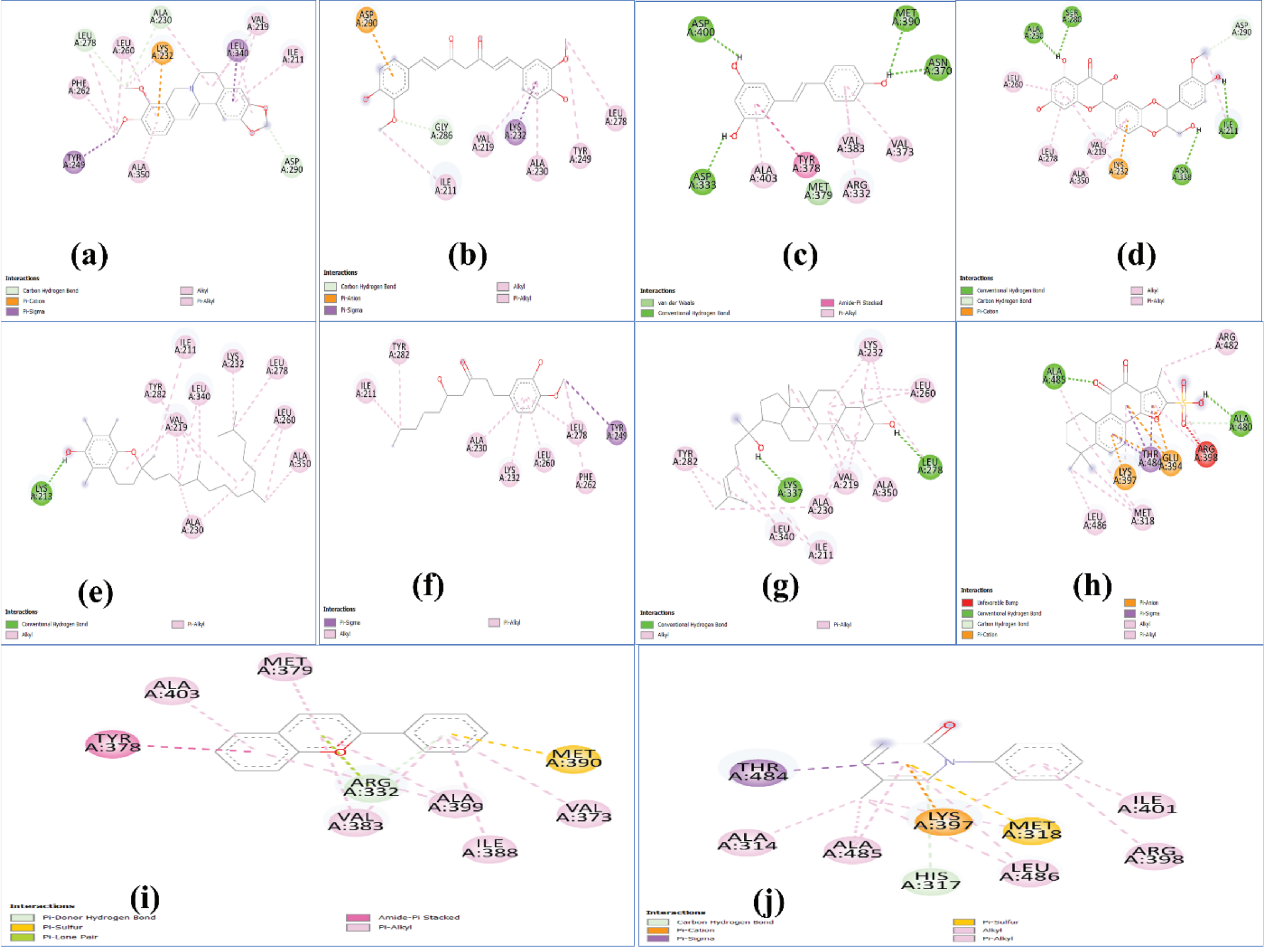
2D interaction plots of **(a)** berberine, **(b)** curcumin, **(c)** resveratrol, **(d)** silymarin, **(e)** tocopherol, **(f)** ginger, **(g)** ginseng, **(h)** danshen, **(i)** anthocyanin, and **(j)** pirfenidone with TGF-β protein. TGF-β, transforming growth factor-beta.

**Figure 2.**
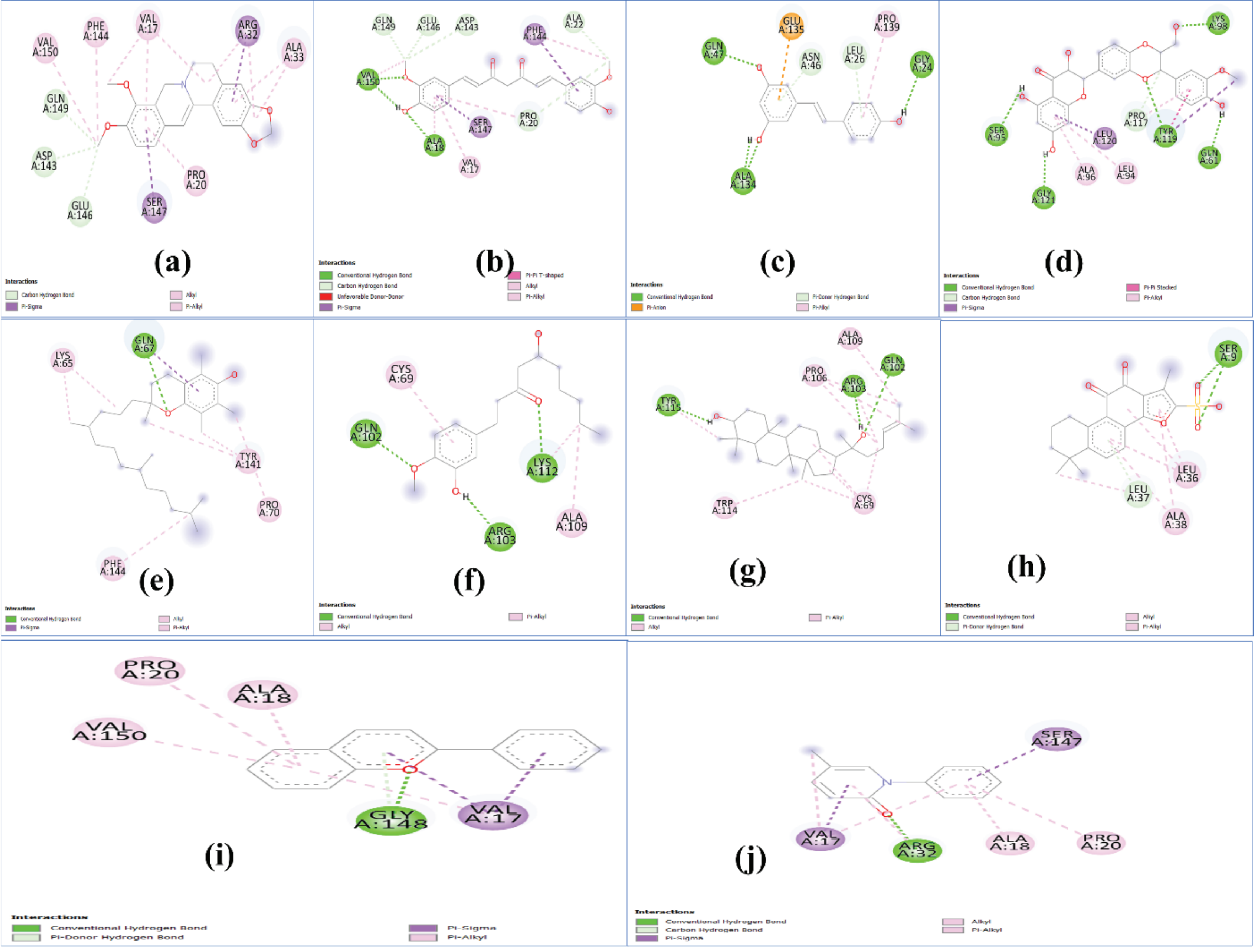
2D interaction plots of **(a)** berberine, **(b)** curcumin, **(c)** resveratrol, **(d)** silymarin, **(e)** tocopherol, **(f)** ginger, **(g)** ginseng, **(h)** danshen, **(i)** anthocyanin, and **(j)** pirfenidone with TNF-α. TNF-α, tumor necrosis factor-alpha.

The 2D interaction plots showing the interactions of different ligands with TGF-β and TNF-α proteins are shown in Figures 1 and 2, respectively. All the important parameters such as the number of hydrogen bonds, binding energy, binding affinity, and different interactions were taken into account and are summarized in Tables 1 and 2. Moreover, inhibition coefficients, which are crucial for understanding the binding affinities and potential efficacy of the compounds, have been included to provide a clearer understanding of the results. Hydrophilic hydrogen bonds, hydrophobic interactions (pi–pi, pi–cation, and pi–anion), and the polar interactions between two oppositely charged ions were taken into consideration while screening of the herbal drug. Indeed, of all, hydrogen bonds are one of the most crucial bonds in drug binding due to their high specificity, absorption, and metabolism.

**Table 1.** The results of in silico docking study for different ligands with TGF-β

Protein	Ligand	No. of hydrogen bonds	Interaction energy (kcal/mol)	Inhibition coefficient (μm)	Prominent bonds
TGF-β	Berberine	0	–8.21	0.953	Pi cation, pi sigma, pi alkyl
	Curcumin	0	–6.06	36.41	Pi cation, pi sigma, pi alkyl
	Resveratrol	4	–8.66	0.447	Hydrogen bond, Amide pi stacked
	Silymarin	4	–8.09	1.18	Hydrogen bond, Pi cation
	Tocopherol	1	–8.41	0.689	Hydrogen bond, pi alkyl
	Ginger	0	–5.46	99.52	Pi sigma, pi alkyl
	Ginseng	2	–10.83	0.011	Hydrogen bond, pi alkyl
	Danshen	2	–11.58	0.003	Hydrogen bond, Pi cation, pi anion, pi sigma, pi alkyl
	Anthocyanin	0	–8.73	0.396	Pi donor hydrogen bond, Pi-sulfur, Pi lone pair, amide pi stacked
	Pirfenidone	0	–7.03	7.08	Pi cation, pi sigma, pi sulfur, pi alkyl

1 TGF-β, transforming growth factor-beta.

**Table 2.** The results of in silico docking study for different ligands with TNF-α

Protein	Ligand	No. of hydrogen bonds	Interaction energy (kcal/mol)	Inhibition coefficient (μm)	Prominent bonds
TNF-α	Berberine	0	–7.11	6.11	Pi sigma, pi alkyl
	Curcumin	3	–7.17	5.54	Hydrogen bond, Pi sigma, Pi T shaped
	Resveratrol	4	–5.88	49.02	Hydrogen bond, Pi anion
	Silymarin	5	–7.21	5.17	Hydrogen bond, Pi sigma, Pi stacked
	Tocopherol	1	–5.98	41.16	Hydrogen bond, Pi sigma, pi alkyl
	Ginger	3	–3.91	0.011	Hydrogen bond, pi alkyl
	Ginseng	3	–8.50	0.590	Hydrogen bond, pi alkyl
	Danshen	2	–7.21	5.22	Hydrogen bond, pi alkyl
	Anthocyanin	1	–7.41	3.72	Hydrogen bond, Pi donor hydrogen bond, pi sigma, pi alkyl
	Pirfenidone	1	–6.33	22.81	Hydrogen bond, pi sigma, pi alkyl

1 TNF-α, tumor necrosis factor-alpha.

First, considering the results obtained for TGF-β, danshen and ginseng possess the highest binding energy, that is, -11.58 kcal/mol and -10.83 kcal/mol, and both possess 2 hydrogen bonds. Next, resveratrol and silymarin both possess 4 hydrogen bonds but less energy than the aforementioned drugs, that is, -8.66 kcal/mol and -8.09 kcal/mol. Moreover, tocopherol, berberine, and anthocyanin possess comparable binding energy to curcumin, that is, -8.41 kcal/mol, -8.21 kcal/mol, and -8.73 kcal/mol, but they possess 1, 0, and 0, respectively, in terms of the number of hydrogen bonds. Curcumin and ginger possess the least TGF-β inhibition activity because of zero hydrogen bonds and -6.06 kcal/mol and -5.46 kcal/mol binding energy. While considering the synthetic drug pirfenidone, it showed zero hydrogen bonds and a binding affinity of -7.03 kcal/mol, which is less than or even comparable with most of the selected herbal molecules except curcumin and ginger.

Second, considering the results obtained for TNF-α, ginseng was found to possess the best activity in terms of both the number of hydrogen bonds and binding energy (-8.50 kcal/mol). Of all, silymarin possesses the maximum number of hydrogen bonds, that is, 5, and a binding energy of -7.21 kcal/mol. Moreover, danshen, anthocyanin, curcumin, and berberine possess comparable binding energy, that is, -7.21 kcal/mol, -7.41 kcal/mol, -7.17 kcal/mol, and -7.11 kcal/mol, and they possess 2, 1, 3, and 0, respectively, in terms of the number of hydrogen bonds. Furthermore, resveratrol and tocopherol possess lesser binding energies compared with the above-stated drugs, that is, -5.88 kcal/mol and -5.98 kcal/mol, with 4 and 1 hydrogen bonds, respectively. Ginger possesses 3 hydrogen bonds, but the binding affinity was found to be less than the minimum -4.5 kcal/mol, that is, -3.91 kcal/mol. While considering the synthetic drug pirfenidone, it showed 1 hydrogen bond and a binding affinity of -6.33 kcal/mol, which is less than or even comparable with most of the selected herbal molecules except ginger and tocopherol.

The study’s findings suggest that the selected herbal compounds, particularly those targeting TGF-β and TNF-α, could be broadly applicable in treating liver fibrosis across different etiologies of chronic liver disease (Table 3). However, the efficacy of these compounds may vary depending on the specific etiology, given the distinct pathophysiological mechanisms involved. Further research, including in vitro and in vivo studies specific to each etiology, is necessary to confirm these effects and to optimize treatment strategies for different types of chronic liver disease.

**Table 3.** The different etiologies of chronic liver disease in the context of fibrogenesis

	Pathogenesis	Study implications
Viral hepatitis (Hepatitis B and C)	Viral hepatitis is a major cause of chronic liver inflammation and fibrosis. The hepatitis viruses directly infect hepatocytes, leading to immune-mediated liver damage, chronic inflammation, and subsequent fibrogenesis. TGF-β is known to play a crucial role in the fibrogenic response to viral infection by promoting the activation of HSCs and ECM deposition.	In the context of viral hepatitis, the inhibition of TGF-β by the herbal compounds identified in our study (e.g., danshen and ginseng) could be particularly beneficial in reducing fibrosis progression. TNF-α inhibition might also help mitigate the chronic inflammatory response triggered by viral infection, thus potentially slowing down the fibrogenic process [35–37].
ALD	Chronic alcohol consumption leads to liver injury through oxidative stress, lipid peroxidation, and the production of pro-inflammatory cytokines, including TNF-α. Alcoholinduced activation of HSCs and the resulting ECM deposition are key contributors to fibrosis in ALD. The role of TGF-β in promoting fibrogenesis is also prominent in ALD.	The compounds that target TNF-α, such as ginseng, may help reduce inflammation and hepatocyte apoptosis associated with ALD [38]. Additionally, targeting TGF-β could be effective in mitigating the progression of fibrosis in ALD by reducing HSC activation and ECM accumulation [39].
NAFLD and NASH	NAFLD and NASH are increasingly recognized as leading causes of liver fibrosis. The pathogenesis involves insulin resistance, lipid accumulation in hepatocytes, oxidative stress, and chronic inflammation. TGF-β plays a pivotal role in driving fibrosis in NASH by inducing the transformation of HSCs into myofibroblasts. TNF-α also contributes to inflammation and hepatocyte injury in NAFLD/NASH.	The findings suggest that the herbal compounds with strong TGF-β inhibitory effects, such as danshen, may be particularly effective in treating fibrosis in NAFLD/NASH [40]. Additionally, TNF-α inhibition by compounds like silymarin could help alleviate inflammation and slow down the progression to fibrosis [41].
AIH	AIH is characterized by immune-mediated attack on hepatocytes, leading to chronic inflammation and fibrosis. The persistent activation of immune cells and the production of cytokines, including TNF-α, are central to the pathogenesis of AIH. TGF-β is also involved in the fibrotic response in AIH.	Herbal compounds that inhibit TNF-α and TGF-β may offer therapeutic benefits in AIH by reducing immune-mediated inflammation and subsequent fibrosis. The dual inhibition of these pathways could potentially slow the progression of fibrosis and improve liver function in patients with AIH [42, 43].

1 AIH, autoimmune hepatitis; ALD, alcohol-related liver disease; ECM, extracellular matrix; HSCs, hepatic stellate cells; NAFLD, non-alcoholic fatty liver disease; NASH, non-alcoholic steatohepatitis; TGF-β, transforming growth factor-beta; TNF-α, tumor necrosis factor-alpha.

## Discussion

The results of this study indicate that herbal compounds like danshen and ginseng exhibit significant anti-fibrotic potential due to their strong binding affinities with TGF-β and TNF-α. These findings align with previous studies that underscore the central role of TGF-β as a master regulator in the fibrotic process, influencing everything from the initial inflammatory response to the activation of HSCs and subsequent collagen deposition. Similarly, the role of TNF-α in promoting liver inflammation, apoptosis, and necroptosis, all precursors to fibrosis, further supports its selection as a primary target in this study. The interactions of TNF-α with other signaling molecules and pathways emphasize its significant role in the progression from inflammation to fibrosis.

Our findings not only support the use of these herbal compounds in managing liver fibrosis but also highlight the advantage of their multi-targeted approach, which contrasts with the more limited scope of single-target synthetic drugs like pirfenidone. The detailed analysis of binding interactions and inhibition coefficients provided in this study offers a clearer picture of the efficacy of these herbal compounds.

While TGF-β and TNF-α were chosen for their well-established relevance in liver fibrosis and therapeutic potential, it is important to acknowledge that other pathways, such as platelet-derived growth factor (PDGF), mitogen-activated protein kinase (MAPK), mitogen-activated protein kinase/ERK kinase (MEK), mammalian target of rapamycin (mTOR), and protein kinase B (AKT), are also critical in the fibrogenic process. These pathways are involved in cell proliferation, survival, and metabolism, contributing to the complex network of signaling events that drive fibrosis. Future studies should extend the investigation to these additional pathways to provide a more comprehensive understanding of the antifibrotic potential of the selected herbal compounds. This could involve computational screening and experimental validation targeting these pathways.

Given the complexity of fibrosis, a multi-target therapeutic approach that modulates several of these pathways may be more effective. This approach could involve combining compounds that target different pathways or developing single compounds that can modulate multiple signaling cascades.

The primary strength of this study lies in its comprehensive analysis of multiple herbal compounds across two critical fibrotic pathways (TGF-β and TNF-α). However, the limitations include the reliance on in silico methods, which may not fully capture the complex biological interactions in vivo. Future studies should aim to validate these findings through experimental models and clinical trials to ensure the translational potential of these herbal compounds.

This in silico study provides key insights into the potential anti-fibrotic effects of various herbal compounds, particularly those targeting TGF-β and TNF-α proteins. Ginseng and danshen exhibited the strongest binding affinities, indicating their potential as effective agents in managing liver fibrosis. Other compounds, like silymarin, berberine, and anthocyanin, also showed promising activity, though to a lesser extent. However, the study’s reliance on computational screening alone limits the ability to draw firm conclusions about the efficacy and safety of these compounds in a biological context. The in silico approach, while useful for initial screening, cannot fully capture the complex biological processes involved in fibrosis or predict the dynamic effects of these compounds in vivo. Therefore, further validation through in vitro and in vivo studies, as well as clinical trials, is essential to confirm the anti-fibrotic potential of these herbal drugs. Additionally, the study did not investigate other crucial pathways involved in fibrogenesis, such as PDGF, MAPK, MEK, mTOR, and AKT, which could provide a more holistic understanding of these compounds’ anti-fibrotic actions. Future research should address these pathways and consider the effects of these herbal drugs across different chronic liver disease etiologies to develop a more effective therapeutic strategy. In conclusion, while this study highlights the potential of herbal compounds like ginseng and danshen in targeting liver fibrosis, it underscores the need for further research to validate these findings and explore additional mechanisms of action.
